# High throughput discovery of protein variants using proteomics informed by transcriptomics

**DOI:** 10.1093/nar/gky295

**Published:** 2018-04-30

**Authors:** Shyamasree Saha, David A Matthews, Conrad Bessant

**Affiliations:** 1 School of Biological and Chemical Sciences, Queen Mary University of London, Mile End, London E1 4NS, UK; 2 School of Cellular and Molecular Medicine, University of Bristol, University Walk, Bristol BS8 1TD, UK; 3 Centre for Computational Biology, Life Sciences Initiative, Queen Mary University of London, Mile End, London E1 4NS, UK

## Abstract

Proteomics informed by transcriptomics (PIT), in which proteomic MS/MS spectra are searched against open reading frames derived from *de novo* assembled transcripts, can reveal previously unknown translated genomic elements (TGEs). However, determining which TGEs are truly novel, which are variants of known proteins, and which are simply artefacts of poor sequence assembly, is challenging. We have designed and implemented an automated solution that classifies putative TGEs by comparing to reference proteome sequences. This allows large-scale identification of sequence polymorphisms, splice isoforms and novel TGEs supported by presence or absence of variant-specific peptide evidence. Unlike previously reported methods, ours does not require a catalogue of known variants, making it more applicable to non-model organisms. The method was validated on human PIT data, then applied to *Mus musculus, Pteropus alecto* and *Aedes aegypti*. Novel discoveries included 60 human protein isoforms, 32 392 polymorphisms in *P. alecto*, and TGEs with non-methionine start sites including tyrosine.

## INTRODUCTION

RNA sequencing (RNA-Seq) followed by *de novo* transcript assembly provides unprecedented insight into gene expression in a given sample, even if the species under study has a poorly annotated genome (1). However, an assembled transcript might not correspond to a functional protein, either for biological reasons or because of sequencing or assembly errors. To resolve this ambiguity we developed the PIT (proteomics informed by transcriptomics) methodology in which spectra acquired from liquid chromatography tandem mass spectrometry (LC–MS/MS) proteomics are searched against open reading frames (ORFs) derived from *de novo* assembled transcripts acquired from the same sample (2). Using sample-specific ORFs allows unbiased identification of translated genomic elements (TGEs), unlike traditional proteomics where spectra are searched against reference protein sequences. PIT therefore allows discovery of new TGEs, including variants of known proteins, and can provide confirmation of transcriptomic observations.

While we have previously published software pipelines for PIT analysis (3), their output is essentially a list of identified TGE sequences (i.e. ORFs supported by peptide evidence). Further post-processing is needed to confidently classify each TGE identification and the sample-specific events that underpin them, such as single amino acid polymorphisms (SAPs), insertions and deletions (INDELs) and alternative splicing. Such events have been associated with disease phenotypes (4–8) and gene regulation (9–11). The significance of single nucleotide polymorphisms (SNPs) in disease phenotypes has prompted several studies to confirm SAPs using mass-spectrometry data (12–14). Many alternative splice isoforms have been observed using RNA-Seq (13,15) but it is unclear how many are translated. A common method for confirming variations at protein level has been to search spectra against a reference proteome augmented with an existing database of known variations or variations identified from RNA-Seq data, although not necessarily from the same sample (13,16–20). The disadvantage of relying on existing databases is that novel protein variants cannot be found—a particular limitation for non-model organisms where databases are incomplete or unavailable.

**Figure 1.**
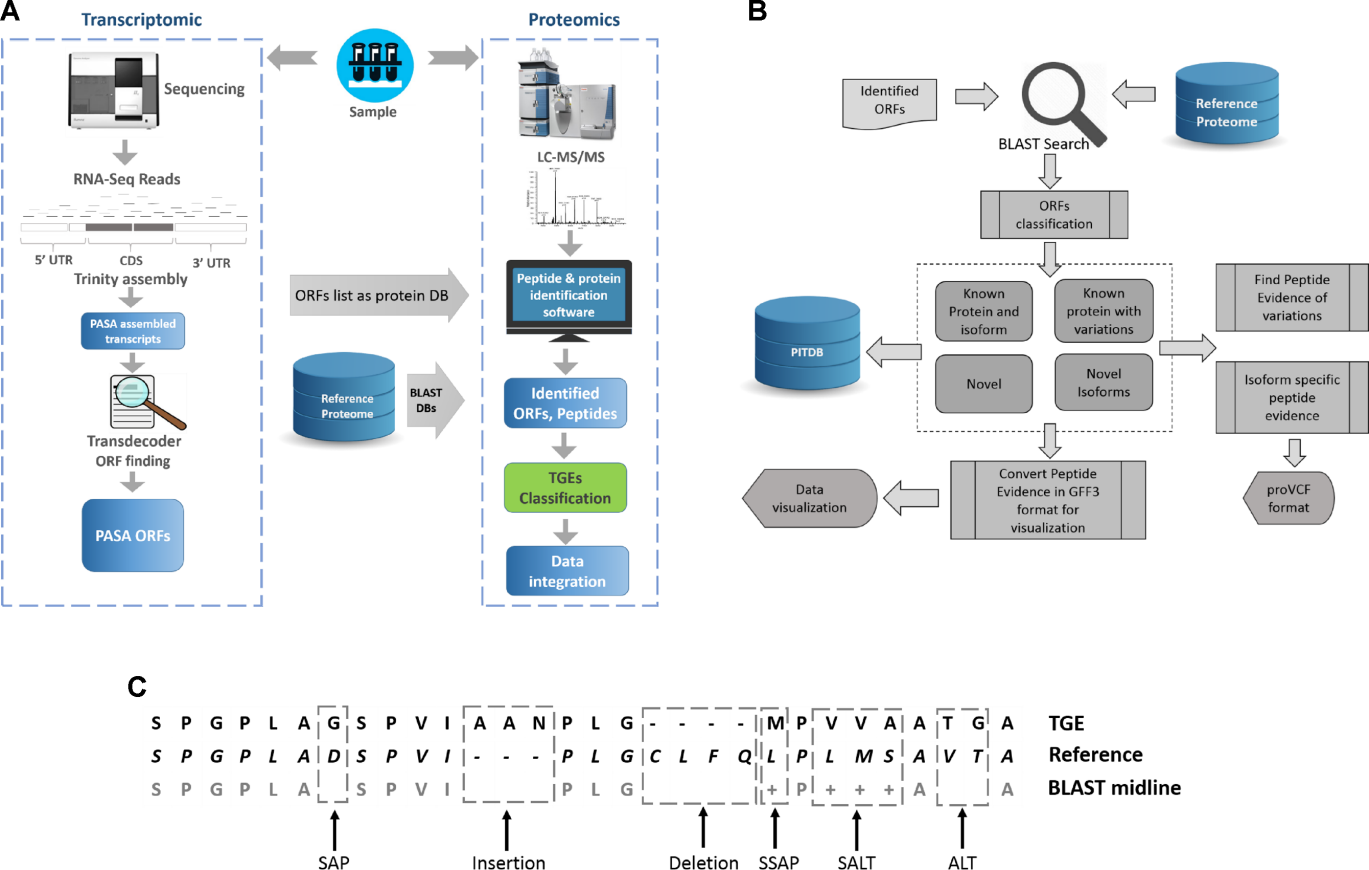
PIT pipeline, now including classification of TGEs. (**A**) RNA-Seq assembly begins with the Trinity *de novo* transcript assembler. PASA is then used to assemble spliced alignments and identify alternative splicing events at transcript level. Transdecoder is used to predict ORFs from PASA’s transcripts. (**B**) TGEs are classified, based on sequence similarity to an existing proteome, into four main classes, (i) known protein or isoform, (ii) known protein or isoform with polymorphisms, (iii) novel isoforms and (iv) novel TGE. Within these main classes there are four polymorphism categories and sixteen novel isoform classes. We look for supporting peptides from the mass spectrometry (MS) data to verify these events at protein level. Variation information and peptide evidence for all the identified TGEs is output and deposited in a database called PITDB. (**C**) Nomenclature of polymorphism types derived from BLAST alignments used in this paper—see main text for details.

Here, we present a TGE classification pipeline that generates variation information directly from RNA-Seq data for each sample, and seeks to confirm this at peptide level using proteomics data from the same sample (Figure 1A and B). The result is a molecular survey of unprecedented detail, with TGEs simultaneously classified into groups including novel proteins, known proteins, protein isoforms and proteins with SAPs and other polymorphisms.

## MATERIALS AND METHODS

### Data

The pipeline was evaluated on data acquired from a human (HeLa) cell line infected with adenovirus (2), then applied to data from three other experiments: *Pteropus alecto* kidney cell line PaKiT03 exposed to Nelson Bay orthoreovirus (NBV) (21), *Mus musculus* fibroblast L929 cell line (22), and immortalised *Aedes Aegypti* cell line Aag2 (23). Details of the proteomics and RNA-Seq data acquisition, and information about where to find this data, are summarised in Supplementary Table S1. The results generated by applying our pipeline to the data are available in the specially created database PITDB (21) [http://pitdb.org] (experiment accession numbers EXP000001, EXP000003, EXP000004 and EXP000008).

### RNA-Seq transcript assembly and protein identification

RNA-Seq reads were initially assembled *de novo* using Trinity (24). Default Trinity read trimming was used along with ‘trimmomatic’ and ‘normalize_reads’ for quality control. Clusters of overlapping Trinity transcripts were assembled into maximal alignment assemblies using the Program to Assemble Spliced Alignments (PASA) (25). PASA runs the seqclean (26; https://sourceforge.net/projects/seqclean/) tool to discard low quality sequences, find evidence of polyadenylation, strip poly-A tails and trim vectors. It then maps remaining transcripts to a reference genome using a spliced alignment process that infers the intron-exon structure of the parent gene. Reference genomes used were hg38 (human), mm10 (*M. musculus*), ASM32557v1 (*P. alecto*) and aedes-aegypti-liverpoolscaffoldsaaegl3 (*A. aegypti*). Any transcripts that do not map to the selected genome assembly (e.g. from viruses) are discarded at this stage. Applying PASA reduces the number of incomplete ORFs and duplicate transcripts, minimising search space in subsequent peptide identification (sSupplementary Figure S1).

Transdecoder (27) was then used for six frame translation of the transcripts, using the default universal genetic code in which methionine is the start codon. Transdecoder assigns ORFs to one of four classes: complete, 5prime_partial, 3prime_partial and internal, based on existence of start and stop codon in the transcript (Supplementary Figure S2). Missing start or stop codons may be due to poor sequence assembly, or alternative start/stop codons. One transcript may produce multiple ORFs, and transcripts with identical protein coding regions (but different untranslated regions) can produce identical ORFs. Duplicate ORFs are retained prior to protein identification to preserve transcript relationships, but are merged into a single TGE when reporting results.

MS-GF+ (28) was used for peptide spectrum matching, followed by mzidentML-lib (29) for FDR calculation, thresholding, and protein grouping. MS-GF+ computes PSM and peptide level q-value using a target-decoy (30) approach. The search database for each sample contained the ORFs obtained from the RNA-Seq data for that sample, plus contaminant sequences from the common Repository of Adventitious Proteins (http://www.thegpm.org/crap). We used 1% global PSM-level FDR and only ORFs with at least two identified peptides were retained as TGEs.

### Classification of observed TGEs by ORF homology

TGEs were classified according to their sequence similarity to a reference proteome using BLAST. BLOSUM80 substitution matrix was used as it is best suited for comparing closely related sequences (31). UniProt complete proteomes were used as the reference for all species except *A. aegypti*, for which a superior proteome was taken from VectorBase. TGEs with 100% sequence identity to a reference protein are classed as known protein, or known protein isoform if the sequence is flagged as an isoform in the reference database. A TGE that does not map to a reference protein with a BLAST *e*-value below 1 × 10^−30^ is classified as novel.

#### Polymorphic proteins

TGEs are classed as known protein with polymorphism when the BLAST alignments are not identical but have e-value below 1 × 10^−30^ and cover the full length of the TGE and the reference protein by introducing polymorphisms such as SAPs, alterations (polymorphisms involving multiple AAs—labeled as ALT), insertions and deletions. Similarity of polymorphisms is identified from the BLAST alignment midline string, as shown in Figure 1C. If an AA is replaced by a chemically similar AA, we call it a SSAP (similar SAP). The same applies to alteration events: an ALT where all AAs have similar chemical properties to their reference sequence counterpart is assigned to a separate category called similar alteration (SALT).

#### Isoform classification

**Figure 2.**
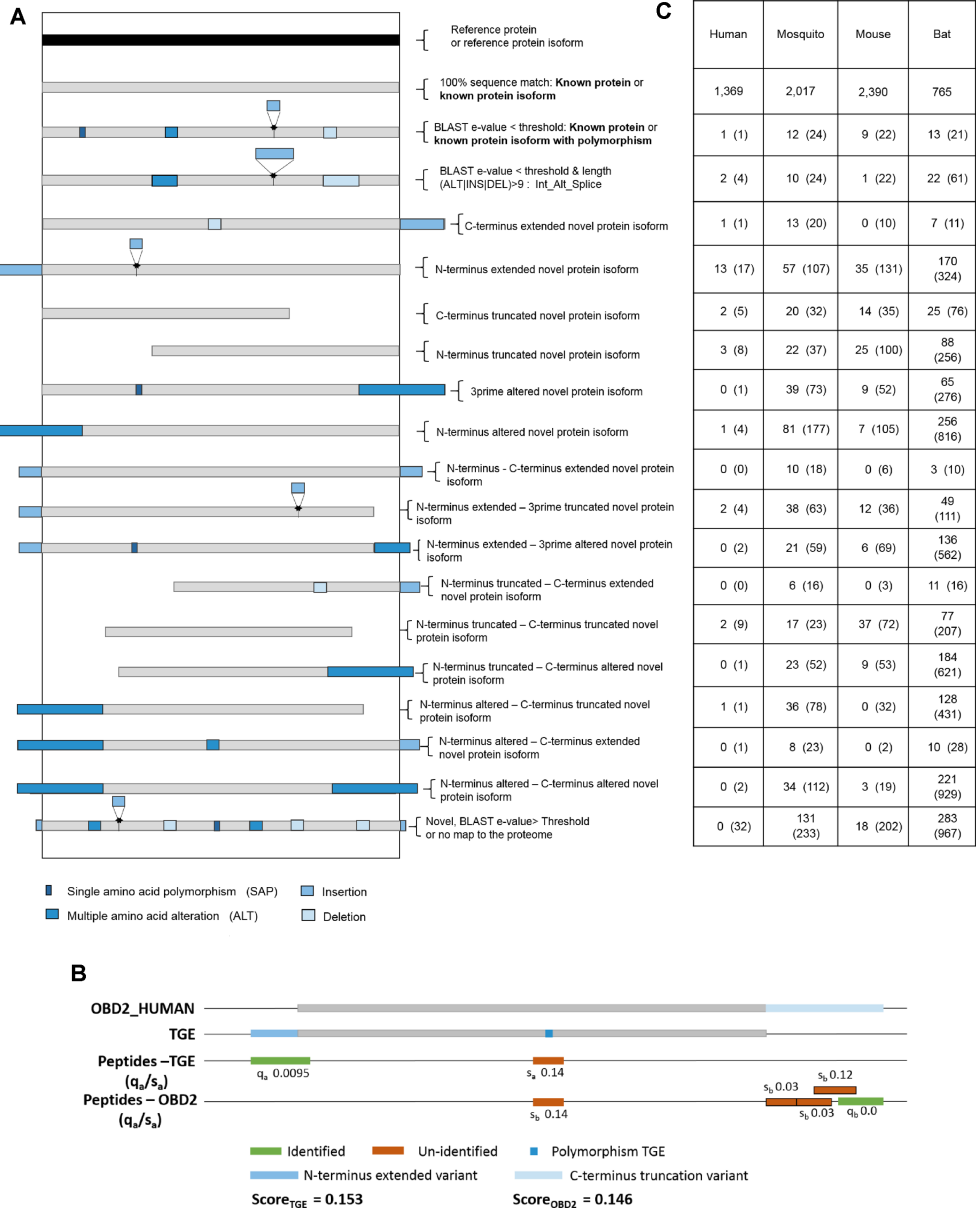
(**A**) Classification of TGEs based on BLAST alignment to UniProt proteome (including isoforms) of the species under study. TGEs with 100% sequence map to UniProt proteins are labelled as known proteins or known isoforms. TGEs with BLAST *e*-value above 1 × 10^−30^ or that do not map to a UniProt protein are classified as novel. The remaining TGEs are classified into one of 17 types based on location, length and type of variation. (**B**) Example of scoring a putative novel isoform based on its mapping to the most homologous protein found by BLAST (ODB2_HUMAN). Only peptides not shared between the TGE and reference are used to compute the scores of the TGE and the reference protein, using Equation (1). *Q*-value scores and sample-specific detectability scores (SS) are used for identified and unidentified peptides respectively. In this case, the TGE score exceeds that of the reference sequence, suggesting we have a novel variant of ODB2. (**C**) Identified TGEs of each type supported by peptide evidence, and unique peptide evidence in parentheses. The proportion of novel findings is higher in species with less well annotated genomes.

Polymorphisms involving more than nine AAs are assigned to a separate internal alternative splice variant group (labeled SV), accounting for alternative splicing events such as exon skipping, intron retention and mutually exclusive exons. The nine AA threshold is generally accepted as the shortest length of an exon (∼99% of protein coding exons are longer than 27 bp). Some TGEs do not map to the full length of the reference protein, or they extend beyond the reference protein. They may also contain polymorphisms. These TGEs are putative novel isoforms, which we categorize into fifteen different classes depending on the nature and location of the variation, as shown in Figure 2A. These classes describe variations at the N-terminal (5-prime end), the C-terminal (3-prime end), or both ends, of the TGE.

### Confirmation of protein variants using peptide evidence

Applying the aforementioned classification strategy to TGEs in this study resulted in the majority being classified as putative novel isoforms and variant proteins. Evidence underpinning each TGE observation was at least two peptide observations mapped to the ORF, but not necessarily to variations within the ORF. Many supposedly novel variants could therefore be due to RNA-Seq errors or poor transcript assembly affecting regions of the ORF not covered by peptide identifications. To separate these from true TGE observations, two methods were applied – a simple approach that demands variant-specific peptides, and a probabilistic scoring approach in which the likely presence of a variant is computed with respect to the reference alternative.

#### Variant-specific peptides

Unlike previous studies that rely on prior knowledge of SNPs and splice sites, we identify variations within our TGE classification pipeline and check for peptides mapping specifically to the variant areas of the TGEs on a sample by sample basis. The majority of these peptides are shared with other TGEs in the sample, so we report peptides uniquely mapping to the variation region separately as this is stronger evidence of the variation.

#### Scoring of variants using predicted peptide detectability

Given that the sequence coverage of LC-MS/MS proteomics is generally low (e.g. ∼17% for known human proteins in this study) it is arguably too conservative to demand peptide evidence for every variant. Some variants are covered by a single peptide, which may not be detectable by MS. A more advanced strategy was therefore implemented, in which predicted peptide detectability, together with peptide identification confidence (represented by q-value), is used to determine the probability that a novel protein variant is more likely to be present in the sample than its corresponding reference protein.

We used an enhanced version of CONSeQuence (32) to calculate a sequence-based detectability score for every tryptic peptide that could be identified in the sample (as trypsin was used for proteolysis in all samples), then calibrated these to a sample-specific detection score (*s*) using a transform function built using empirical peptide detectability information from ORFs in the sample that had already been identified as known proteins. By comparing the combined probability of detection of the set of peptides, *R* = {*r*_1_, *r*_2_ … *r*_*n*_}, that uniquely describe the reference protein against the set of peptides, *V* = {*v*_1_, *v*_2_ … *v_m_*}, that describe the protein variant it is possible to predict which is most likely to be present in the sample. The details of this calculation are shown in Equation (1), and an example of its use is shown in Figure 2B.

(1) \begin{equation*}\begin{array}{@{}*{1}{l}@{}} {scor{e_{{\rm{variant}}}} = \frac{1}{{\left| V \right| + \left| R \right|}}\left( {\sum\limits_{\forall a \in A} {(1 - {q_a})} } \right.\ \ }\\ {\left. {-\sum\limits_{\forall b \in B} {\frac{{1 - {q_b}}}{4}} + \sum\limits_{\forall b \in B\prime } {\frac{{{s_b}}}{8}} - \sum\limits_{\forall a \in A\prime } {\frac{{{s_a}}}{8}} } \right)} \end{array}\end{equation*}

where *A* is the set of identified peptides from *V, B* is the set of identified peptides from *R, A*′ is the set of unidentified peptides in *V* and *B*′ is the set of unidentified peptides in *R*.

An equivalent equation is used to compute *score*_reference_

(2) \begin{equation*}\begin{array}{@{}*{1}{l}@{}} {scor{e_{{\rm{reference}}}} = \frac{1}{{\left| R \right| + \left| V \right|}}\ \left( {\mathop \sum \limits_{\forall b \in B} (1 - {q_b})} \right.}\\ {\left. {-\mathop \sum \limits_{\forall a \in A} \frac{{1 - {q_a}}}{4} + \mathop \sum \limits_{\forall a \in A^\prime} \frac{{{s_a}}}{8} - \mathop \sum \limits_{\forall b \in B^\prime} \frac{{{s_b}}}{8}} \right)} \end{array}\end{equation*}

The score, *score*_variant_, for the TGE is calculated by considering only peptides that cover variant regions of the protein. Scores are assigned to each of these peptides as follows. Peptides from the TGE are given a score of 1 – *q* (where *q* is the lowest *q*-value for that peptide) if they are identified in the sample or –*s*/8 if they are not identified. Peptides from the reference sequence are given a score of (1 – *q*)/4 if they are identified in the sample or –(*s*/8) if they were not identified. The sum of peptide scores for the reference sequence is then subtracted from the sum of peptide scores for the TGE and normalised for peptide count to give the final TGE score. A similar equation is used to calculate *score*_reference_ (Equation 2). The denominators of unidentified peptides were set to 8 to compensate for an anticipated LC-MS/MS peptide coverage of 12.5%. The denominator of 4 is used to ensure that the difference between *score*_variant_ and *score*_reference_ is small when both reference and variant-specific peptides are observed, indicating that both sequences are likely to exist in the sample.

The *score*_variant_ and *score*_reference_ are calculated separately so that the magnitude of the difference between them can be used to accommodate situations where both versions of the sequence may be present. Applying a threshold to this difference can separate confidently classified variants from reference proteins. Unless otherwise stated, we report TGEs as variants when *score*_variant_ > *score*_reference_. More detail regarding the scoring pipeline can be found in Supplementary Figure S3.

#### Validation of variant scoring method using human data

PIT data was processed in the absence of prior protein variation information (i.e. TGE classification BLASTed against the UniProt canonical proteome only) such that all observed isoforms would be classified as novel isoforms (Supplementary Figure S4). Separately, TGE classification was performed by BLASTing against the UniProt human proteome including known isoforms. Comparing the list of novel isoforms from the first classification with the list of identified known isoforms from the second classification indicated the ability of the classification pipeline to identify isoforms in the absence of prior knowledge.

### Rating TGEs by available evidence

The overall confidence in the presence of an individual TGE can be assessed by considering all the aforementioned evidence collectively. For example, a list of observed TGEs can be ranked using a rating system such as that shown in Supplementary Table S2, where higher ratings are awarded to TGEs with more rigorous forms of evidence such as a unique PSM covering a variant region. This allows identified TGEs to be prioritised for further evaluation or validation.

## RESULTS AND DISCUSSION

### TGE classification

Results for all four species are summarised in Table 1, with sample-specific breakdowns provided in Supplementary Tables S3-S5. Prior to considering variation-specific peptide evidence, the majority of putative TGEs are classified as novel isoforms, or known proteins with sequence polymorphism. Only 39% of putative TGEs from the human dataset have 100% sequence similarity to reference protein, a proportion that is lower still for *A. aegypti* (36%), *M. musculus* (10%) and *P. alecto* (∼3%). For *M. musculus* and *P. alecto*, PIT identifies significantly more sequences compared to the standard database search, probably due to PIT’s ability to account for sample specific variations. However, the number of protein variations is likely to be a significant overestimate as the TGEs are not necessarily supported by variant-specific peptide evidence at this stage.

**Table 1.** Overview of PIT TGE classification results

Dataset (number of samples in parentheses)				*Homo sapiens* (1)	*Mus musculus* (8)	*Pteropus alecto* (9)	*Aedes aegypti* (1)
Total spectra				210,560	293,894	350,890	829,093
Standard search	Peptides			24,187	23,151	22,554	58,336
	PAGs (protein ambiguity groups)			3,011	3,536	3,270	4,743
	Total proteins			12,589	14,107	3,522	5,692
	SwissProt	Canonical		3,302	3,534	2	71
		Isoform		3,365	1,344	0	79
	TrEMBL			5,922	9,229	3,520	5,542
PIT search	Peptides			21,612	24,297	23,875	52,221
	PAGs			2,646	2,814	2,701	4,394
	Total TGEs			3,504	24,602	28,311	5,488
	TGEs mapping to SwissProt	Canonical	Total	1,134	1,270	0	77
			Complete ORF	1,134	1,268	0	77
		Isoform	Total	197	195	0	1
			Complete ORF	197	193	0	1
	TGEs mapping to TrEMBL		Total	38	925	765	1,939
			Complete ORF	38	915	756	1,930
	Putative novel isoform	SwissProt	Total	1,815	12,351	0	57
			Complete ORF	174	1,864	0	20
			Score	363	707	0	9
			With specific peptide evidence	50	357	0	11
			With unique specific peptide ev.	24	76	0	7
		TrEMBL	Total	233	9,194	26,328	3,080
			Complete ORF	30	1,643	5,700	1,077
			Score	92	488	5,092	891
			With specific peptide evidence	10	390	4,735	903
			With unique specific peptide ev.	3	82	1,452	428
	Known protein with	SwissProt	Total	47	278	0	4
	polymorphism		Complete ORF	21	92	0	4
			Score	7	14	0	0
			With specific peptide evidence	1	6	0	0
			With unique specific peptide ev.	1	3	0	0
		TrEMBL	Total	8	187	251	97
			Complete ORF	5	86	95	85
			Score	0	31	25	32
			With specific peptide evidence	0	16	21	24
			With unique specific peptide ev.	0	6	13	12
	Novel TGE		Total	32	202	967	233
			Complete ORF	3	38	236	61
			With unique peptide evidence	0	18	283	131

To allow comparison with standard proteomics methods, peptide and protein identification was also performed for each species by searching directly against the reference proteome—the results of this are shown in the top (standard search) portion of the table. Throughout the table, identified proteins are shown based on the source reference sequence: Swiss-Prot or TrEMBL. Swiss-Prot proteins are further divided into two groups, canonical and isoform. TGEs with exact sequence map to reference proteins are classed as known proteins. TGEs not mapping to any reference proteins or with e-value above the threshold are classified as novel TGEs. The remaining TGEs are classified as known proteins with polymorphism, or novel isoforms of known proteins. The novel isoform TGEs are further separated into 16 classes and reliability of this annotation is verified by isoform-specific peptide evidence (see Supplementary Table S5 for details). Peptide and protein counts reported in the table are unique sequences across all the samples for datasets with multiple samples and average PAG (protein ambiguity group) counts are reported for these cases.

### Variations supported by simple peptide evidence

The proportion of variations with variant-specific peptide evidence varied greatly among species. Only a small minority of those in Table 1 have isoform specific peptides (∼2% for human and *M. musculus*; ∼20% for *P. Alecto* and *A. Aegypti*, reflecting the relative annotation quality of these species). The distribution of identified isoforms with variant-specific evidence among the various types is summarised in Figure 2C (numbers in parentheses indicate TGEs that meet the more conservative criteria of having unique peptide evidence). Besides identifying peptides from the variant regions, we found junction peptides for TGEs from alternative and extended isoform classes. The majority of these junction peptides support alternative sequence variations. Many of these junction peptides are also unique peptides (33–40% for the non-human datasets). This demonstrates that the PIT pipeline is capable of high throughput discovery of novel isoforms in the absence of prior information about gene structure.

**Figure 3.**
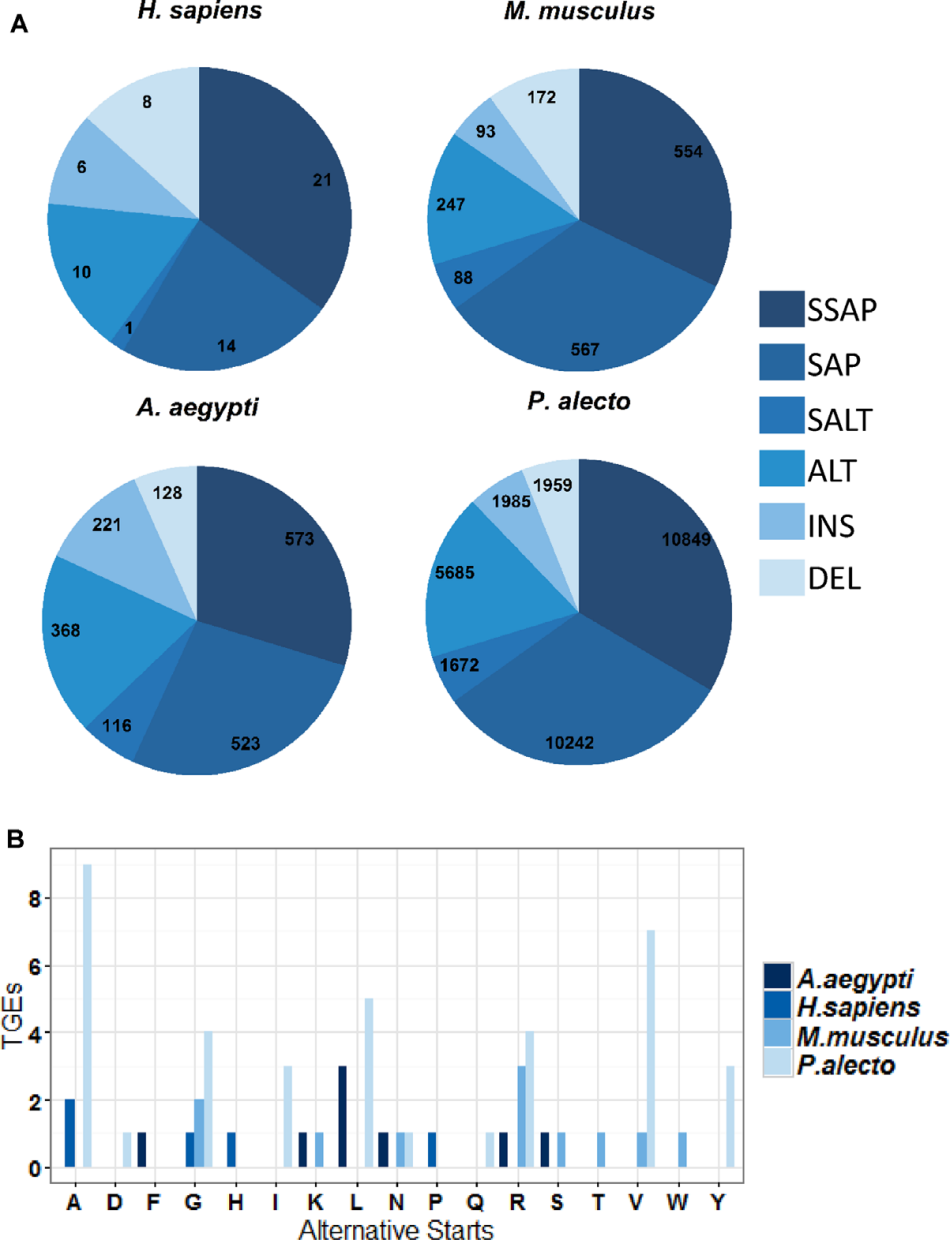
(**A**) Overview of polymorphisms with variation-specific peptide evidence, for each species. (**B**) Distribution of alternative start codons confirmed by peptide evidence for different species. For context, 172 and 166 Swiss-Prot proteins have alternative starts for human and *M. musculus* respectively. Swiss-Prot contains only two proteins for *P. alecto*, and both start with valine.

Regarding polymorphisms, human and *M. musculus* have the lowest percentage of peptide-supported polymorphisms, only 5% and 7% respectively, whereas *A. aegypti* and *P. alecto* have 15% and 23%. These were found in all variant TGE classes, and in known proteins (counted separately in Table 1). Peptide supported polymorphisms are shown for each species in Figure 3A. The total number of polymorphisms range from just 60 for human, through to 32 392 for *P. Alecto*, reflecting the relative quality of the reference proteomes for these species. This suggests significant scope for improving the *P. Alecto* reference proteome, by using the polymorphisms identified by PIT to correct existing protein sequences predicted from an imperfect reference genome.

### Alternative start codons

The majority of TGEs classified as known protein were from ORFs classified as complete by Transdecoder (see Table 1), suggesting that many incomplete ORFs are due to poor sequence assembly. However, some proteins do not start with methionine so Transdecoder incompleteness does not necessarily indicate an erroneous ORF. Alternative start codons are found in Swiss-Prot for all the species in this study except *A. aegypti*. Our results include several non-methionine starts with peptide evidence (often unique peptide evidence) for all the species in this study (Figure 3B). To avoid the possibility that an alternative start is called due to the N-terminus of a truncated ORF coinciding with a tryptic cleavage site, we discounted all TGEs with alternative starts where the reference protein has lysine or arginine at the preceding position. The highest number of non-methionine start codons supported by peptide evidence are observed for *P. alecto*, most of which are valine or alanine. We identified TGEs with N-terminus methionine removed, which is significant for function and stability (33).

### Validation of variant scoring method using human data

**Figure 4.**
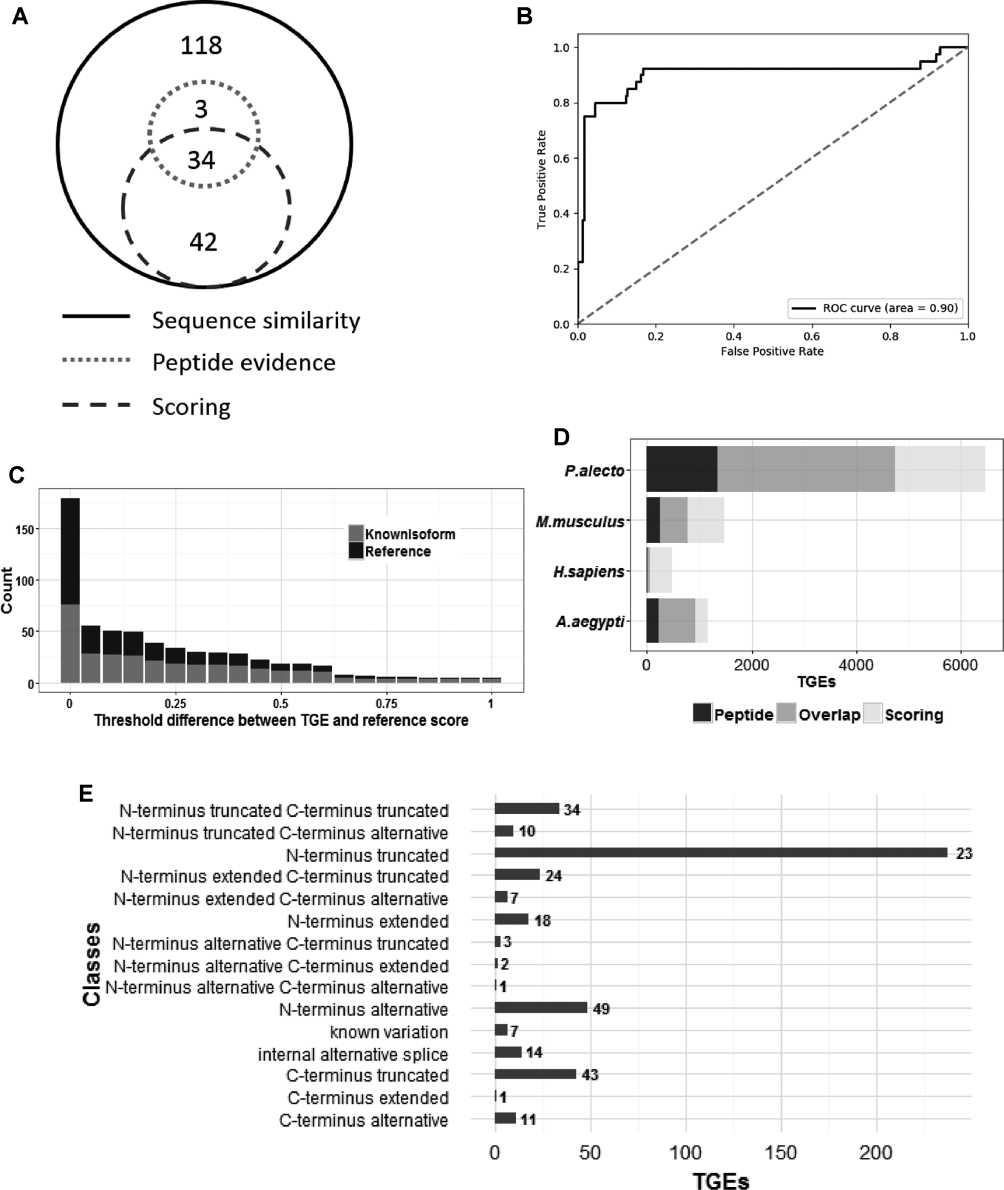
TGE scoring and validation. (**A**) Comparison of methods for confirming the identification of novel protein isoforms, applied to human PIT data. A set of 197 known isoforms found in the sample using PIT was used for validation. The scoring method can identify 76 of these as isoforms, which is an improvement over the 37 confirmed by the simple peptide evidence approach. (**B**) ROC curve showing performance of the scoring method in comparison to the traditional variant-specific peptide evidence based method for known isoforms. (**C**) The number of TGEs classified as isoform rapidly decreases as the threshold between TGE and reference score is increased, while the proportion of those that have been confirmed as isoforms present in the sample increases. (**D**) Comparison of isoform classification techniques applied on novel isoforms from all species. The scoring method predicts higher numbers of variant isoforms in the sample compared to the peptide evidence method, but misses some TGEs confirmed by peptide evidence. (**E**) Class distribution of TGEs confirmed by the scoring method for novel isoforms in human.

Our scoring-based classification method identified 76 known isoforms of the 197 found to be present in the sample during the evaluation process. These known isoforms are supported by at least two peptides but, as in any proteomics experiment, their presence in the sample cannot be proven definitively without laboratory validation. The scoring method identifies 42 known isoforms that the simple peptide evidence approach missed, but fails to classify three known isoforms reported by the simple peptide evidence approach (Figure 4A). These missed isoforms were due to peptide identifications from the reference having higher confidence than from the variant, and failure to observe highly detectable variant-specific peptides. A ROC curve (Figure 4B) shows the performance of the scoring method using known isoforms identified from the PIT search. Novel protein variants confirmed by variant-specific peptides is the best way to confirm their presence in the sample without separate laboratory validation, therefore we used known isoforms confirmed by variant-specific peptide evidence as the gold standard for the validation process and observed an area under the curve (AUC) of 0.90. Figure 4C shows how increasing the score difference threshold reduces the number of TGEs classified, but increases the proportion of those confirmed as isoforms present in the sample. Protein ambiguity group analysis shows that known isoforms usually share peptides with other variants of the protein, making their presence ambiguous. This evaluation exercise shows that the scoring method can significantly increase the number of confident novel isoform identifications compared to the simple variation-specific peptide evidence approach.

### Scoring variants using predicted peptide detectability

For the human dataset, application of the scoring method with a zero threshold suggests that 455 out of 2048 putative novel isoforms are indeed novel isoforms, and seven out of 55 putative known proteins with polymorphisms are also confirmed (Supplementary Figure S5). As in the evaluation, the scoring method classifies more TGEs than the simple variant-specific peptide evidence method, and there is a significant overlap between the two methods (Figure 4D). Except for one TGE each from *P. alecto* and human, the remaining TGEs supported by junction peptides were classified as variants using the scoring method. Most TGEs confirmed exclusively by the scoring method come from the N-terminus truncated class (see Figure 4E), due to non-identification of highly detectable peptides from the truncated region. In summary, applying the TGE scoring method has allowed us to promote several thousand putative protein variants (14% of the total) to a higher level of confidence.

### Shared TGEs among species

**Figure 5.**
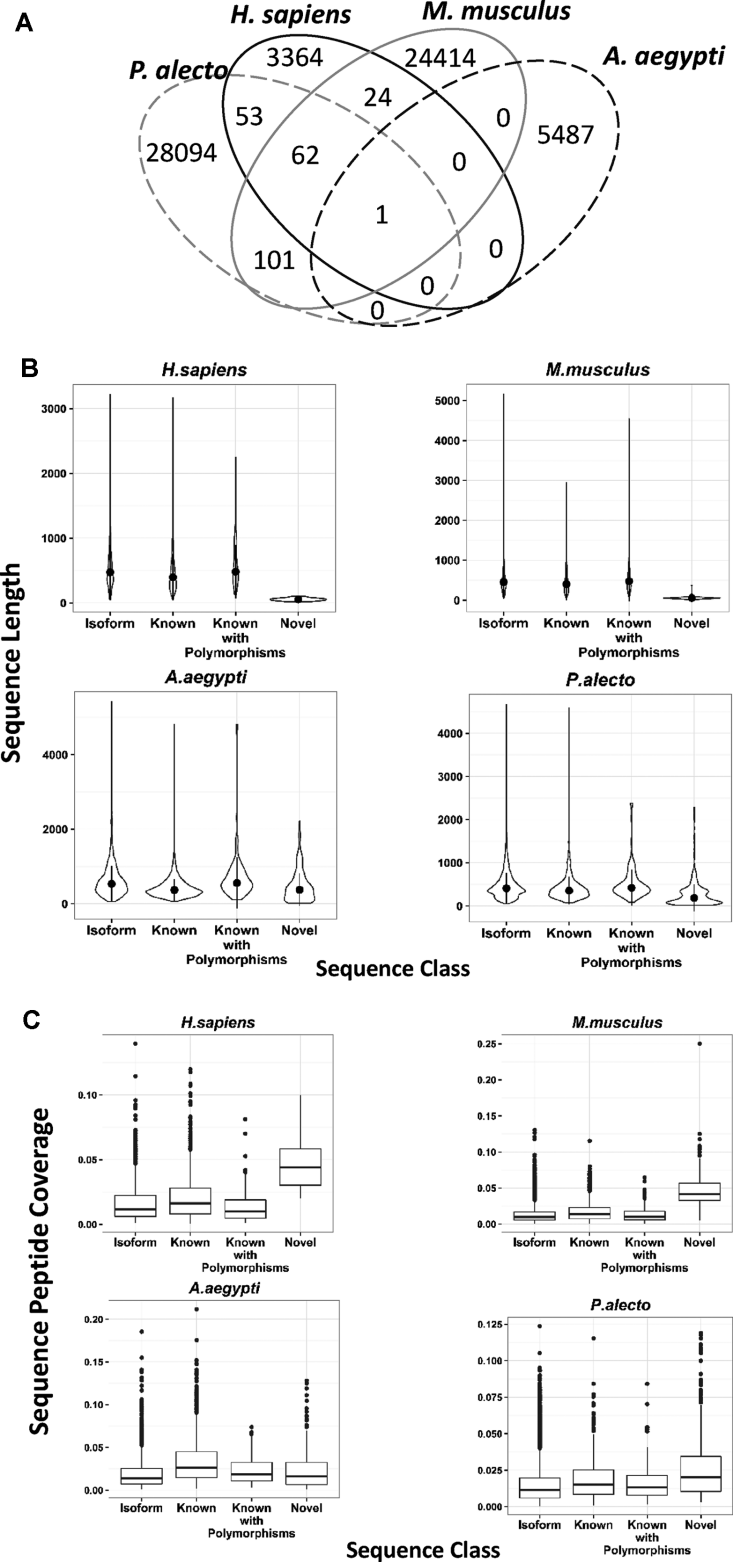
(**A**) Overlap of all identified TGEs across organisms (to be classified as an overlap the TGEs were required to have identical sequences). Overlapping TGEs are often known in one species but novel variant for the others. Some of the overlapping novel variants have variant-specific peptide evidence. (**B**) Length distribution of different TGE classes identified from human, *P. alecto, M. musculus* and *A. aegypti* datasets. Novel TGEs are significantly shorter than the rest of the TGE types for human and mouse, while *A. aegypti* and *P. alecto* have novel TGEs with lengths similar to those of the other TGE classes. (**C**) Distribution of peptide coverage per TGE for different TGE classes in each species.

Only one TGE, histone H3 protein, is observed in all four species—a Swiss-Prot protein for human and *M. musculus* that is reported in TrEMBL for *P. Alecto* and *A. Aegypti*. However, there are many TGEs in common between pairs of species (Figure 5A), most of which are known proteins. Some shared TGEs are classified as known in one species but as novel isoform in another, for example three TGEs that are known *M. musculus* proteins but have been classified as N-terminus truncated (two TGEs) and known protein with polymorphisms for human. The known protein with polymorphism (Vesicle-trafficking protein SEC22b) has unique peptide evidence for one of the polymorphisms. Human also shares 116 identified TGEs with *P. Alecto*, although none of these shared TGEs is a novel variant supported by peptide evidence. One novel isoform of Heterogeneous nuclear ribonucleoprotein D0 protein with peptide evidence is shared between *P. alecto* and *M. musculus* (with unique peptide evidence in mouse). The distribution of known *M. musculus* proteins shared with *P. alecto* is shown in Supplementary Figure S6. Such identifications of the same TGE in multiple species can increase confidence in the biological validity of that identification, and are also relevant to cross-species studies.

### Novel TGEs

We identified novel TGEs in each dataset, from 32 in human to 967 in *P. alecto*, but the majority are not supported by unique peptide evidence (Table 1). In human and *M. musculus*, most putative novel TGEs are significantly shorter than other TGE classes (Figure 5B). A large portion of novel *P. alecto* TGEs are short but overall have median length close to known proteins. Novel *A. aegypti* TGEs also have similar median length compared to their known counterparts but are not skewed towards shorter TGEs. Peptide coverage is similar or higher for novel TGEs (Figure 5C), giving confidence in these identifications. Collectively, this suggests that most of the novel TGEs from *P. alecto* and *A. aegypti* are likely to be newly discovered proteins, whereas those from *M. musculus* and human may be too short to be functional proteins. This is confirmed by the fact that most of the supposedly novel short human TGEs were found to map directly to subsections of multiple existing proteins. They exceed the BLAST e-value threshold because the significance of individual matches decreases when there are multiple matches, but they are very likely to be ORFs predicted from partially assembled transcripts.

## CONCLUSION

The TGE classification pipeline presented here has been shown to be a significant improvement in PIT methodology, providing deeper insight into human samples, and finding large numbers of confidently identified polymorphisms and novel splice variants in non-model species that can be used to rapidly improve their reference proteomes. For example, strong evidence has been found for hundreds of novel TGEs and protein isoforms in *P. alecto* and *A. aegypti*, including many with alternative start codons. The significant reduction in putative TGEs seen when peptide evidence is considered demonstrates the benefit of using PIT rather than extrapolating translated products from RNA-seq data alone. By developing this pipeline and making it publicly available we give the research community the opportunity to adopt this alternative approach.

## DATA AVAILABILITY

The software pipeline, and documentation, is available via GitHub [https://github.com/bezzlab/TGEClassification]. The results generated, including novel protein sequences, are available in PITDB ([http://pitdb.org] with experiment accession numbers EXP000001, EXP000003, EXP000004 and EXP000008.

## Supplementary Material

Supplementary Data

## References

[1] da Fonseca R.R., Albrechtsen A., Themudo G.E., Ramos-Madrigal J., Sibbesen J.A., Maretty L., Zepeda-Mendoza M.L., Campos P.F., Heller R., Pereira R.J. Next-generation biology: Sequencing and data analysis approaches for non-model organisms. Mar. Geonomics. 2016; 30:3–13. 10.1016/j.margen.2016.04.012 27184710

[2] Evans V.C., Barker G., Heesom K.J., Fan J., Bessant C., Matthews D.A. De novo derivation of proteomes from transcriptomes for transcript and protein identification. Nat. Methods. 2012; 9:1207–1211. 23142869 10.1038/nmeth.2227 PMC3581816

[3] Fan J., Saha S., Barker G., Heesom K.J., Ghali F., Jones A.R., Matthews D.A., Bessant C. Galaxy integrated Omics: Web-based Standards-Compliant workflows for proteomics informed by transcriptomics. Mol. Cell. Proteomics. 2015; 14:3087–3093. 26269333 10.1074/mcp.O115.048777 PMC4638048

[4] Di Fede G., Catania M., Morbin M., Rossi G., Suardi S., Mazzoleni G., Merlin M., Giovagnoli A.R., Prioni S., Erbetta A. A recessive mutation in the APP gene with dominant-negative effect on amyloidogenesis. Science (New York, N.Y.). 2009; 323:1473–1477. 10.1126/science.1168979 PMC2728497 19286555

[5] Skotheim R.I., Nees M. Alternative splicing in cancer: noise, functional, or systematic?. Int. J. Biochem. Cell Biol. 2007; 39:1432–1449. 17416541 10.1016/j.biocel.2007.02.016

[6] Andrews S.J., Rothnagel J.A. Emerging evidence for functional peptides encoded by short open reading frames. Nat. Rev. Genet. 2014; 15:193–204. 24514441 10.1038/nrg3520

[7] Ciriello G., Miller M.L., Aksoy B.A., Senbabaoglu Y., Schultz N., Sander C. Emerging landscape of oncogenic signatures across human cancers. Nat. Genet. 2013; 45:1127–1133. 24071851 10.1038/ng.2762 PMC4320046

[8] Sui Z., Wen B., Gao Z., Chen Q. Fusion-Related host proteins are actively regulated by NA during influenza infection as revealed by quantitative proteomics analysis. PLoS One. 2014; 9:e105947. 25153908 10.1371/journal.pone.0105947 PMC4143309

[9] Francesconi M., Lehner B. The effects of genetic variation on gene expression dynamics during development. Nature. 2013; 505:208–211. 24270809 10.1038/nature12772

[10] Banfai B., Jia H., Khatun J., Wood E., Risk B., Gundling W.E. Jr, Kundaje A., Gunawardena H.P., Yu Y., Xie L. Long noncoding RNAs are rarely translated in two human cell lines. Genome Res. 2012; 22:1646–1657. 22955977 10.1101/gr.134767.111 PMC3431482

[11] Cheetham S.W., Gruhl F., Mattick J.S., Dinger M.E. Long noncoding RNAs and the genetics of cancer. Br. J. Cancer. 2013; 108:2419–2425. 23660942 10.1038/bjc.2013.233 PMC3694235

[12] Cao R., Shi Y., Chen S., Ma Y., Chen J., Yang J., Chen G., Shi T. dbSAP: single amino-acid polymorphism database for protein variation detection. Nucleic Acids Res. 2017; 45:D827–D832. 27903894 10.1093/nar/gkw1096 PMC5210569

[13] Sheynkman G.M., Johnson J.E., Jagtap P.D., Shortreed M.R., Onsongo G., Frey B.L., Griffin T.J., Smith L.M. Using galaxy-P to leverage RNA-Seq for the discovery of novel protein variations. BMC Genomics. 2014; 15:703. 25149441 10.1186/1471-2164-15-703 PMC4158061

[14] Pang C.N., Tay A.P., Aya C., Twine N.A., Harkness L., Hart-Smith G., Chia S.Z., Chen Z., Deshpande N.P., Kaakoush N.O. Tools to covisualize and coanalyze proteomic data with genomes and transcriptomes: validation of genes and alternative mRNA splicing. J. Proteome Res. 2014; 13:84–98. 24152167 10.1021/pr400820p

[15] Ruggles K.V., Tang Z., Wang X., Grover H., Askenazi M., Teubl J., Cao S., McLellan M.D., Clauser K.R., Tabb D.L. An analysis of the sensitivity of proteogenomic mapping of somatic mutations and novel splicing events in cancer. Mol. Cell. Proteomics. 2016; 15:1060–1071. 26631509 10.1074/mcp.M115.056226 PMC4813688

[16] Woo S., Cha S.W., Na S., Guest C., Liu T., Smith R.D., Rodland K.D., Payne S., Bafna V. Proteogenomic strategies for identification of aberrant cancer peptides using large-scale next-generation sequencing data. Proteomics. 2014; 14:2719–2730. 25263569 10.1002/pmic.201400206 PMC4256132

[17] Krasnov G.S., Dmitriev A.A., Kudryavtseva A.V., Shargunov A.V., Karpov D.S., Uroshlev L.A., Melnikova N.V., Blinov V.M., Poverennaya E.V., Archakov A.I. PPLine: an automated pipeline for SNP, SAP, and splice variant detection in the context of proteogenomics. J. Proteome Res. 2015; 14:3729–3737. 26147802 10.1021/acs.jproteome.5b00490

[18] Wang X., Slebos R.J.C., Wang D., Halvey P.J., Tabb D.L., Liebler D.C., Zhang B. Protein identification using customized protein sequence databases derived from RNA-Seq data. J. Proteome Res. 2011; 11:1009–1017. 22103967 10.1021/pr200766z PMC3727138

[19] Wang X., Zhang B. customProDB: an R package to generate customized protein databases from RNA-Seq data for proteomics search. Bioinformatics. 2013; 29:3235–3237. 24058055 10.1093/bioinformatics/btt543 PMC3842753

[20] Ning K., Nesvizhskii A.I. The utility of mass spectrometry-based proteomic data for validation of novel alternative splice forms reconstructed from RNA-Seq data: a preliminary assessment. BMC Bioinformatics. 2010; 11(Suppl. 11):S14. 10.1186/1471-2105-11-S11-S14 PMC3024872 21172049

[21] Wynne J.W., Shiell B.J., Marsh G.A., Boyd V., Harper J.A., Heesom K., Monaghan P., Zhou P., Payne J., Klein R. Proteomics informed by transcriptomics reveals Hendra virus sensitizes bat cells to TRAIL-mediated apoptosis. Genome Biol. 2014; 15:532. 25398248 10.1186/s13059-014-0532-x PMC4269970

[22] Mok L., Wynne J.W., Grimley S., Shiell B., Green D., Monaghan P., Pallister J., Bacic A., Michalski W.P. Mouse fibroblast L929 cells are less permissive to infection by Nelson Bay orthoreovirus compared to other mammalian cell lines. J. Gen. Virol. 2015; 96:1787–1794. 25748429 10.1099/vir.0.000112

[23] Maringer K., Yousuf A., Heesom K.J., Fan J., Lee D., Fernandez-Sesma A., Bessant C., Matthews D.A., Davidson A.D. Proteomics informed by transcriptomics for characterising active transposable elements and genome annotation in Aedes aegypti. BMC Genomics. 2017; 18:101. 28103802 10.1186/s12864-016-3432-5 PMC5248466

[24] Grabherr M.G., Haas B.J., Yassour M., Levin J.Z., Thompson D.A., Amit I., Adiconis X., Fan L., Raychowdhury R., Zeng Q. Full-length transcriptome assembly from RNA-Seq data without a reference genome. Nat. Biotechnol. 2011; 29:644–652. 21572440 10.1038/nbt.1883 PMC3571712

[25] Haas B.J., Delcher A.L., Mount S.M., Wortman J.R., Smith R.K., Hannick L.I., Maiti R., Ronning C.M., Rusch D.B., Town C.D. Improving the Arabidopsis genome annotation using maximal transcript alignment assemblies. Nucleic Acids Res. 2003; 31:5654–5666. 14500829 10.1093/nar/gkg770 PMC206470

[26] TIGR Gene Index group. Seqclean. Accessed on 09 Aug 2016.

[27] Haas B.J., Papanicolaou A., Yassour M., Grabherr M., Blood P.D., Bowden J., Couger M.B., Eccles D., Li B., Lieber M. De novo transcript sequence reconstruction from RNA-seq using the Trinity platform for reference generation and analysis. Nat. Protoc. 2013; 8:1494–1512. 23845962 10.1038/nprot.2013.084 PMC3875132

[28] Kim S., Pevzner P.A. MS-GF+ makes progress towards a universal database search tool for proteomics. Nat. Commun. 2014; 5:5277. 25358478 10.1038/ncomms6277 PMC5036525

[29] Ghali F., Krishna R., Lukasse P., Martínez-Bartolomé S., Reisinger F., Hermjakob H., Vizcaíno J.A., Jones A.R. Tools (Viewer, Library and Validator) that facilitate use of the peptide and protein identification standard format, termed mzIdentML. Mol. Cell. Proteomics. 2013; 12:3026–3035. 23813117 10.1074/mcp.O113.029777 PMC3820921

[30] Elias J.E., Gygi S.P. Target-decoy search strategy for increased confidence in large-scale protein identifications by mass spectrometry. Nat. Methods. 2007; 4:207–214. 17327847 10.1038/nmeth1019

[31] Henikoff S., Henikoff J.G. Amino acid substitution matrices from protein blocks. PNAS. 1992; 89:10915–10919. 1438297 10.1073/pnas.89.22.10915 PMC50453

[32] Eyers C.E., Lawless C., Wedge D.C., Lau K.W., Gaskell S.J., Hubbard S.J. CONSeQuence: prediction of reference peptides for absolute quantitative proteomics using consensus machine learning approaches. Mol. Cell. Proteomics. 2011; 10:M110.003384. 10.1074/mcp.M110.003384 PMC3226394 21813416

[33] Liao Y.D., Jeng J.C., Wang C.F., Wang S.C., Chang S.T. Removal of N-terminal methionine from recombinant proteins by engineered E. coli methionine aminopeptidase. Protein Sci. 2004; 13:1802–1810. 15215523 10.1110/ps.04679104 PMC2279930

