# Supplementary material for: High throughput discovery of protein variants using proteomics informed by transcriptomics
Source: Nucleic Acids Res. 2018 Apr 30;46(10):4893–902. doi: 10.1093/nar/gky295 (PMC6007231; doi:10.1093/nar/gky295)
Supplement: Supplementary Data [file gky295_supp.zip › PIT_variants_supplementary_figures.docx]

**Supplementary Figures**


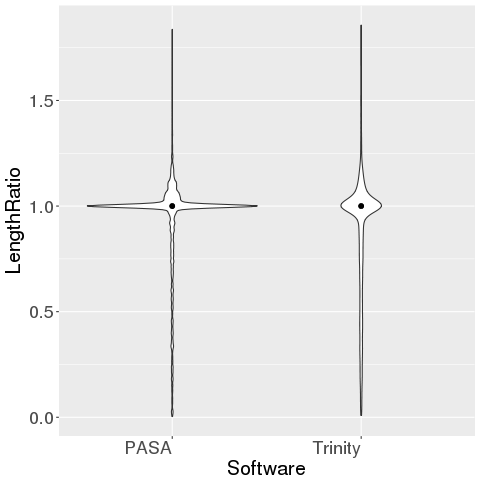


**Figure S1:** Comparison of length of identified TGEs produced from PASA and Trinity assembled transcripts using Transdecoder against the reference proteins that they map to. This violin plot shows that the majority of PASA TGEs are equal in length to the corresponding reference protein. The majority of PASA TGEs are either equal or longer than the corresponding reference proteins. Trinity TGEs are equally distributed on both sides, i.e. shorter and longer ends. We identify more proteins with Trinity assembly because it produces many small transcripts that create short TGEs partially mapping to a UniProt protein, but that protein was not necessarily present in the sample.


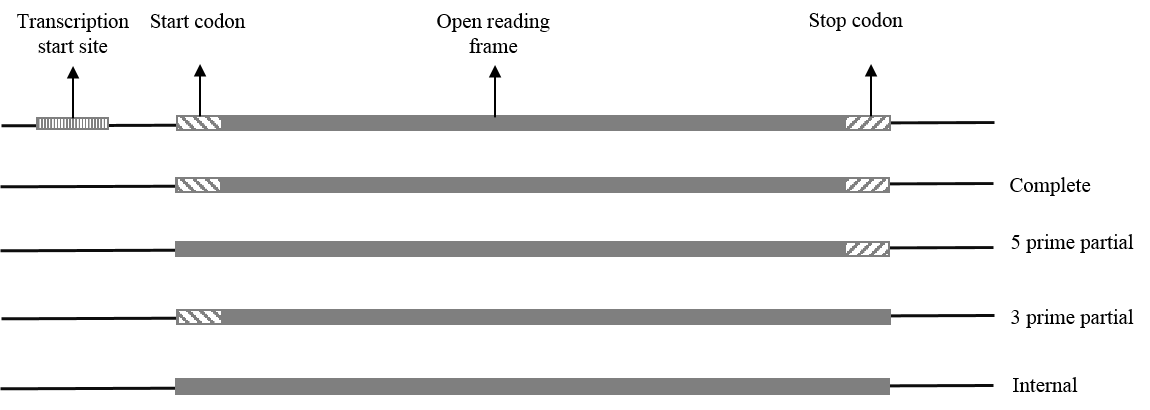


**Figure S2:** Classification of open reading frames depending on presence of start and stop codons. A TGE is called complete when both start and stop codons were found in the transcript. 5’ partial and 3’ partial represents TGEs missing a start or stop codon respectively. A TGE is labelled as internal when both start and stop codons are absent.


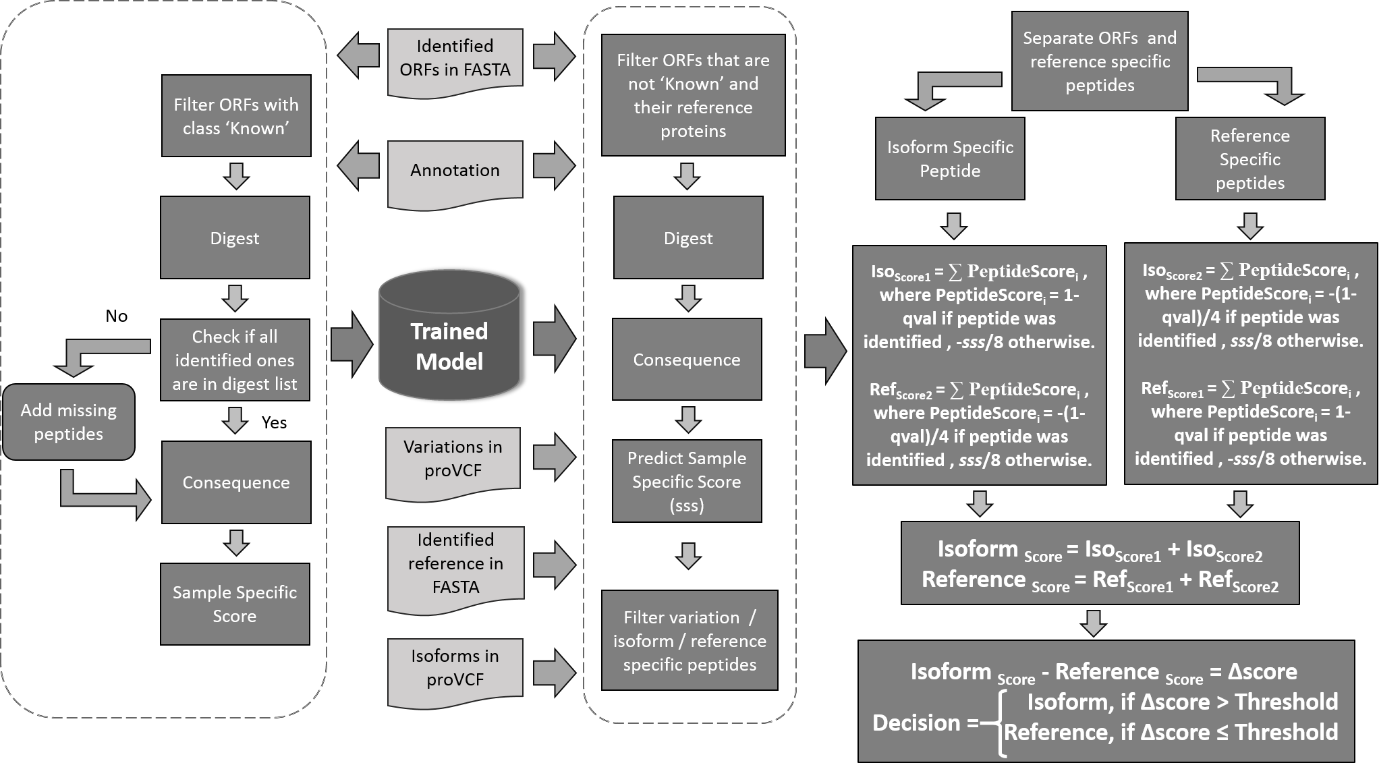


**Figure S3:** Schematic of the variant protein scoring pipeline. We start by mimicking MSGF+ peptide digestion and computing CONSeQuence scores of the peptides. We train a calibration model using peptides from identified TGEs to compute sample specific scores (SS) for each peptide from its consequence scores. We compute the TGE and corresponding reference protein score from the variant-specific peptides using their SS score if the peptide was not identified, or from the q-value if it was identified. TGEs with higher scores compared to the reference proteins are classified as variants.


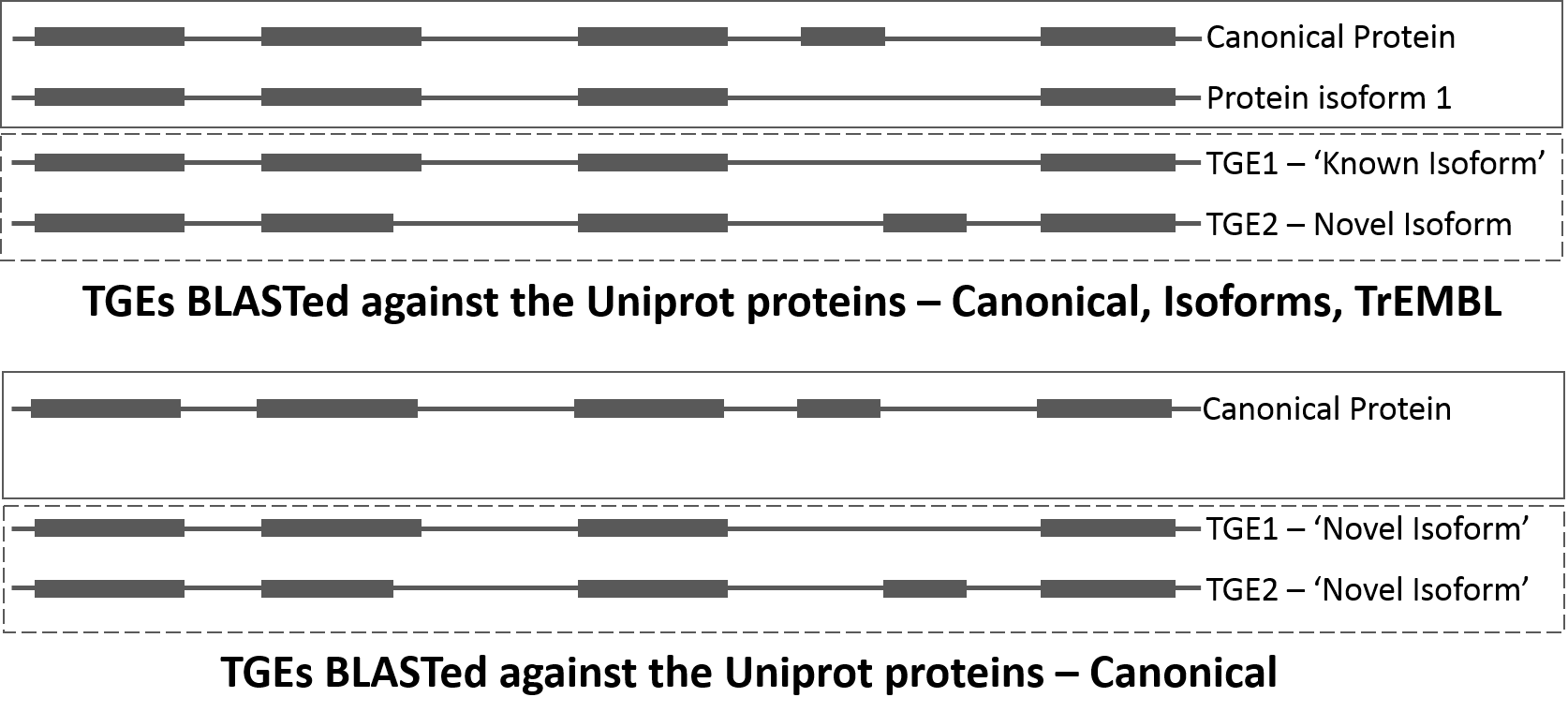


**Figure S4:** To evaluate the scoring method, we BLASTed known isoforms identified in our human experiments against the canonical proteins from UniProt such that they would be classified as novel isoforms using our sequence-based classification method. This figure shows how removal of known isoform sequences from BLAST database forces those sequences to be identified as novel isoforms by our sequence similarity method. We apply our scoring method on these known isoforms to check how many of them are correctly confirmed as variant sequence.


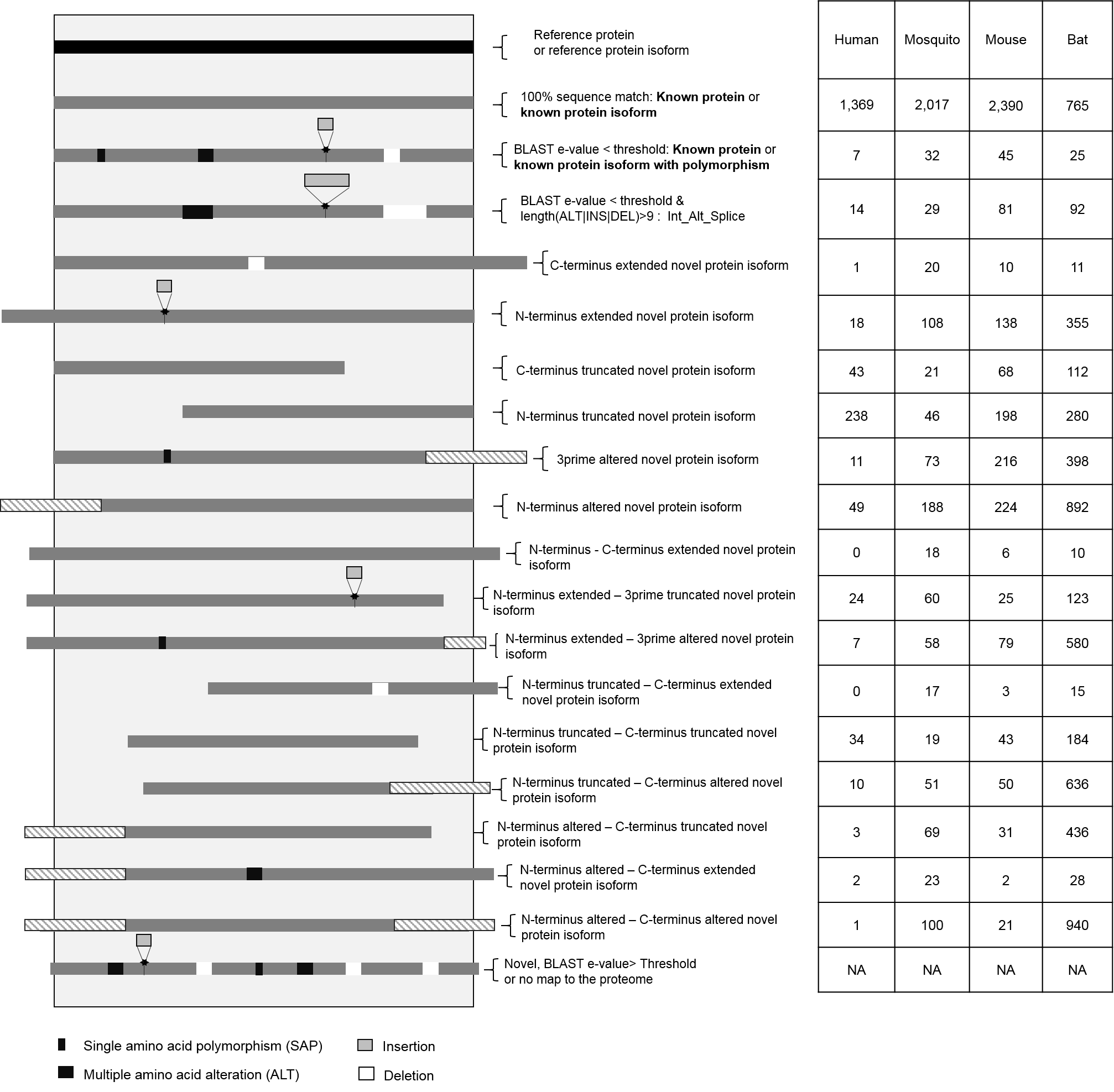


**(b)**

**(a)**

**Figure S5:** (a) Classification of TGEs, as in Figure 2a. (b) A similar similar table to that in Figure 2c, except this shows the number of identified TGEs predicted by the scoring method of each type, for each dataset analysed in this study.


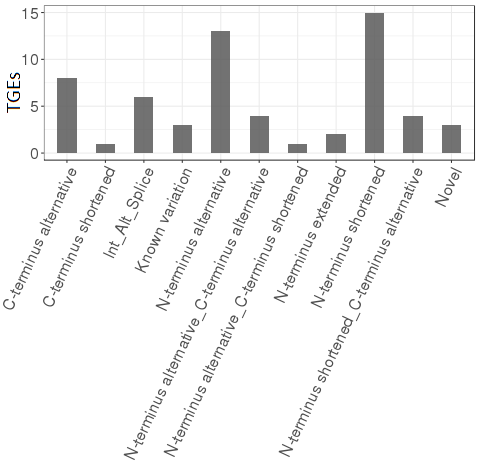


**Figure S6:** TGE class distribution of 60 TGEs mapped to the *P. alecto* proteome that are identified in both *P. alecto* and *M. musculus* experiments. All of these 60 TGEs out of a total of 164 shared TGEs are ‘known’ in respect to the *M. musculus* proteome. Forty two TGEs out of all shared TGEs are known for both species. A similar distribution is observed for TGEs known for human and shared with *P. alecto*. This type of comparison allows identification of novel shared proteins among species.

**
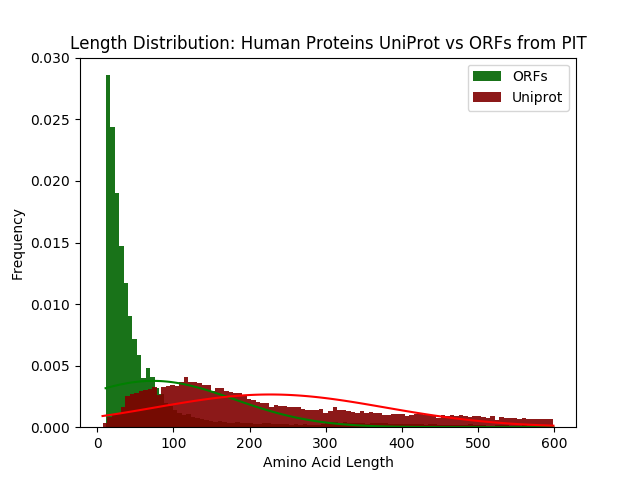
Figure S7:** Length distribution of human UniProt proteins and ORFs predicted from the human RNA-seq data. It shows that Transdecoder produced a lot of small ORFs resulting in skewed length distribution for PIT ORFs.

**
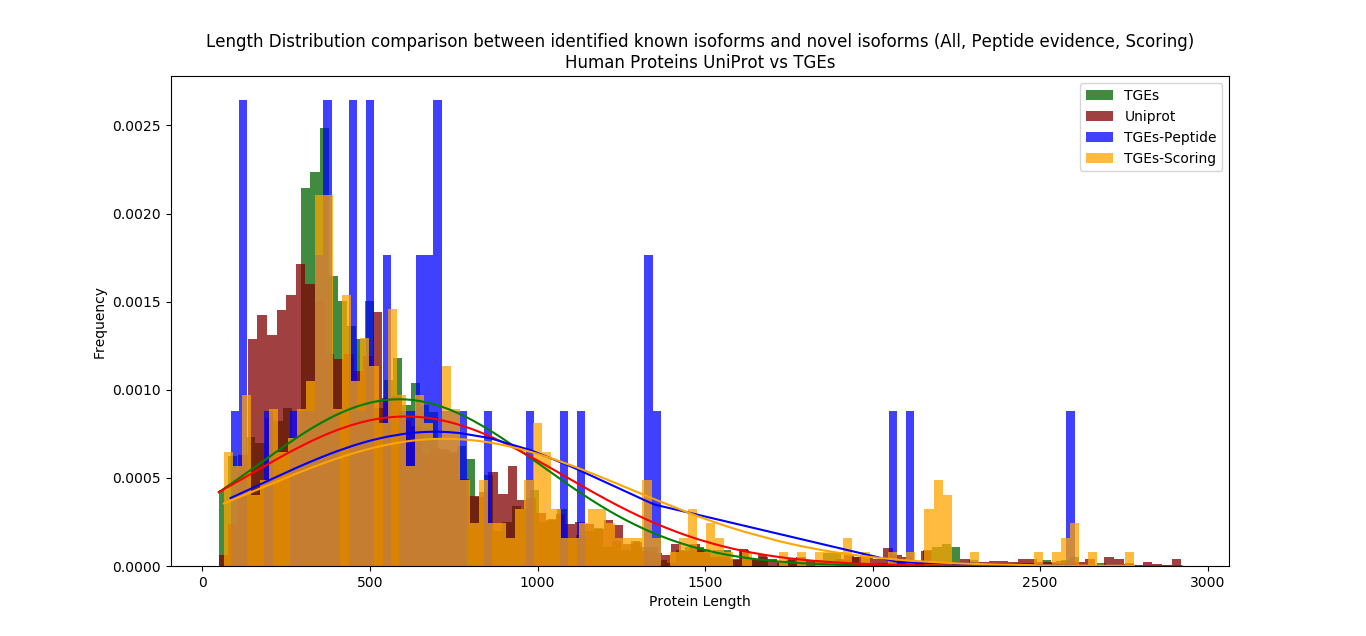
Figure S8:** This figure shows the distributions of identified known human isoforms from a standard proteomics search (searching against UniProt), and novel isoforms from the PIT search with different levels of confirmation, i.e. sequence similary (labelled as TGE), variant specific peptide evidence (TGEs-Peptide) and scoring method (TGEs-Scoring). It shows a similar length distribution among all these groups.
