# Supplementary material for: High throughput discovery of protein variants using proteomics informed by transcriptomics
Source: Nucleic Acids Res. 2018 Apr 30;46(10):4893–902. doi: 10.1093/nar/gky295 (PMC6007231; doi:10.1093/nar/gky295)
Supplement: Supplementary Data [file gky295_supp.zip › TableS2.docx]

**Table S2.** Scheme used to assign confidence ratings to TGE observations that BLAST suggests are variants of known proteins. All TGEs are ORFs derived from transcripts, supported by at least two peptides observed by mass spectrometry. Confidence is increased if the following criteria are met: (a) the ORF is complete (has a recognised start and stop codon), (b) the peptide evidence is from the variant region, (c) the variant peptide(s) uniquely map to this protein and (d) the probability of the variant being genuine is high according to our pipeline.

| Complete ORF | Variant-specific peptide | Unique variant-specific peptide | High probability variant | Rating |
| --- | --- | --- | --- | --- |
| ● | ● | ● | ● | **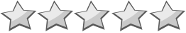** |
| ● | ● |  | ● | **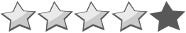** |
| ● | ● | ● |  | **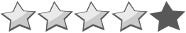** |
| ● | ● |  |  | **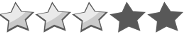** |
|  | ● | ● |  | **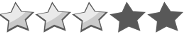** |
|  | ● | ● | ● | **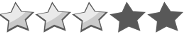** |
|  | ● |  | ● | **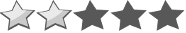** |
| ● |  |  | ● | **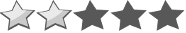** |
|  | ● |  |  | **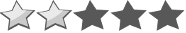** |
|  |  |  | ● | **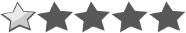** |
| ● |  |  |  | **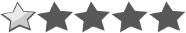** |
|  |  |  |  | **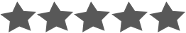** |
